# Recurrent Coronary Vasospasm: A Case of Kounis Syndrome from Anaphylaxis to Contrast Dye

**DOI:** 10.14797/mdcvj.1084

**Published:** 2022-06-28

**Authors:** Katherine Lee Chuy, Proddutur R. Reddy, Aviral Vij

**Affiliations:** 1 Cook County Health, Chicago, Illinois, US; 2 Rush Medical College, Chicago, Illinois, US; 3 Cook County Health, Rush Medical College, Chicago, Illinois, US

**Keywords:** Kounis syndrome, vasospasm, contrast dye, hypersensitivity, anaphylaxis, coronary artery disease

## Abstract

Kounis syndrome is characterized by acute coronary syndrome due to coronary vasospasm or thrombosis following exposure to an allergic stimulus. The presentation can be compounded by cardiovascular collapse due to cardiogenic shock from coronary vasospasm and associated vasodilatory shock from anaphylaxis. A high index of suspicion is crucial for prompt initiation of treatment, which focuses on managing the allergic or anaphylactic process. Here we present a case of coronary vasospasm and anaphylactic shock due to contrast dye exposure during percutaneous coronary intervention of an unstable coronary lesion and its associated diagnostic and therapeutic challenges.

## Introduction

Kounis syndrome (KS) is the occurrence of acute coronary syndromes in the setting of allergic or anaphylactic reactions. Three types of KS have been described based on the presence of coronary vasospasm and atherothrombosis. We present a case of KS due to contrast dye exposure during percutaneous coronary intervention (PCI).

## Case

**Figure 1:**
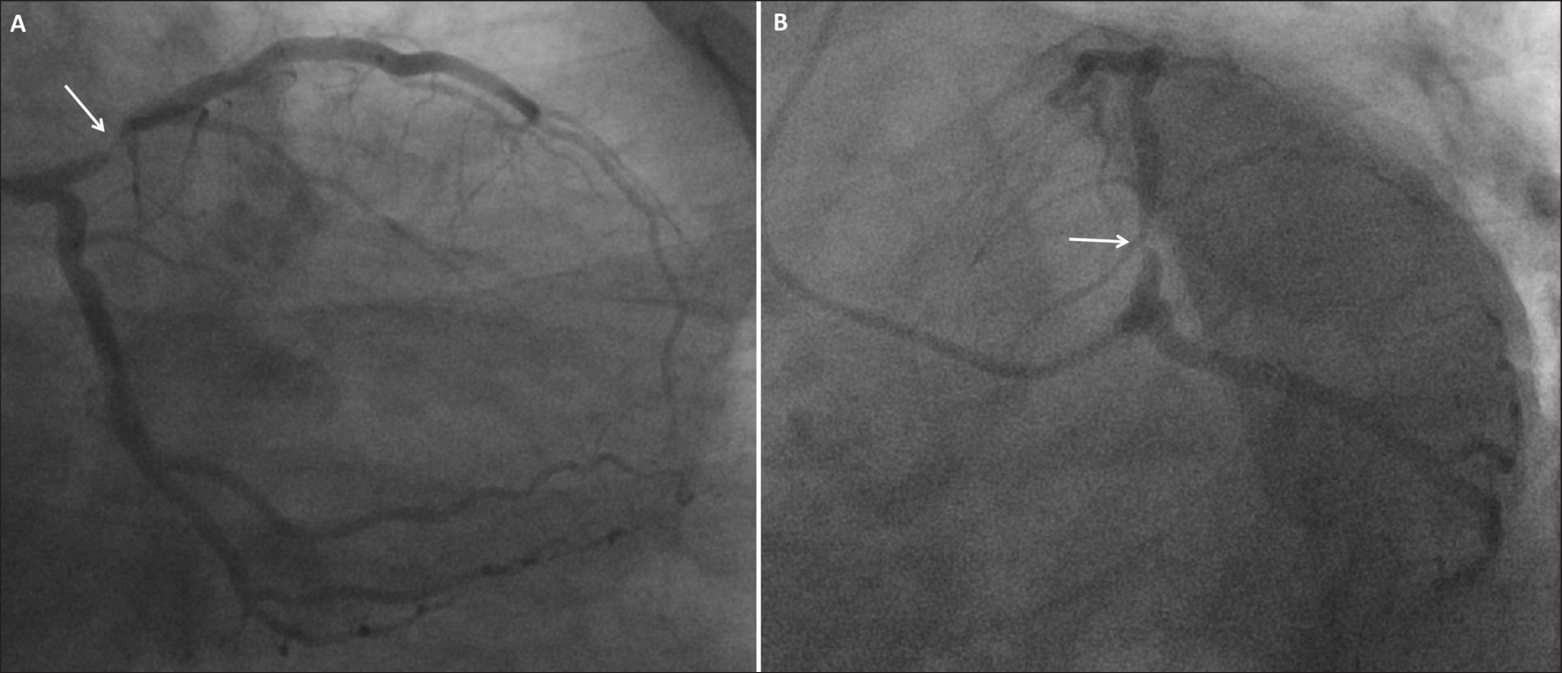
**(A)** RAO caudal view and **(B)** LAO caudal view showing a tubular 90% stenosis in the proximal LAD artery (arrow) and unremarkable LCx artery. RAO: right anterior oblique; LAO: left anterior oblique; LAD: left anterior descending; LCx: left circumflex.

**Figure 2:**
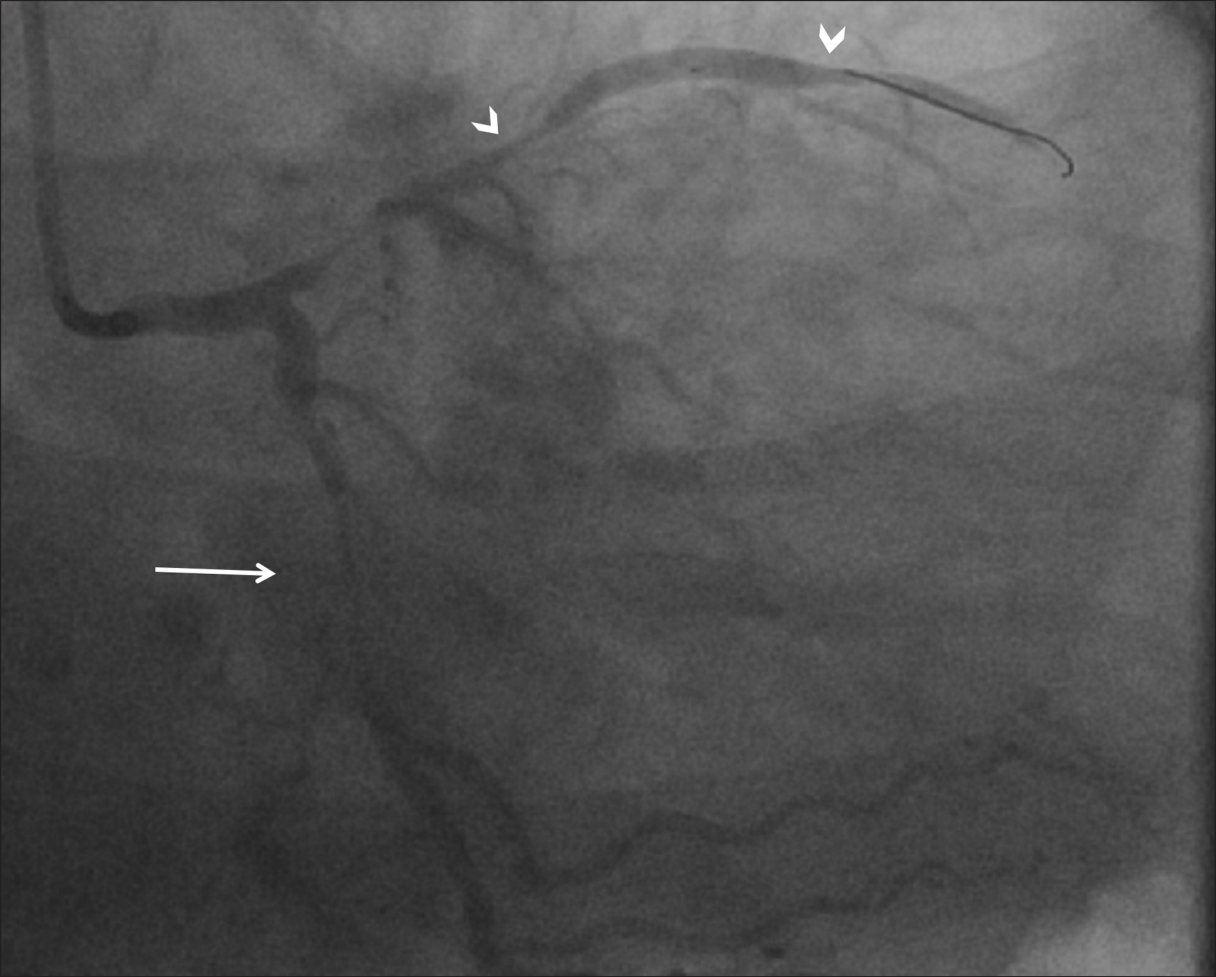
RAO caudal view showing diffuse stenosis in the proximal to mid segment of the previously unremarkable LCx artery with TIMI 2 flow (arrow) and diffuse stenosis in the previously normal mid to distal LAD segments with TIMI 0 flow (arrowheads), consistent with vasospasm. RAO: right anterior oblique; LCx: left circumflex; LAD: left anterior descending; TIMI: thrombolysis in myocardial infarction.

A 59-year-old man with hypertension and type 2 diabetes mellitus presented with unstable angina. Labs that included troponin were within normal limits. Single-photon emission computed tomography (SPECT) myocardial perfusion imaging showed ischemia in the left anterior descending (LAD) artery territory. Subsequently, coronary angiogram revealed a 90% tubular stenosis in the proximal LAD artery (Figure 1; Videos 1, 2, 3). The lesion was crossed with a workhorse 0.014-inch balanced middleweight wire without difficulty. After balloon dilatation of the LAD lesion, we noted loss of flow in the LAD and diffuse spasm in the left circumflex (LCx) arteries with thrombolysis in myocardial infarction (TIMI) 0 and TIMI 2 flow, respectively (Figure 2; Video 4). The patient became hypotensive to 58/34 mm Hg, diaphoretic and complained of chest pressure with evidence of ST elevation on the electrocardiogram. He was started on intravenous fluids and norepinephrine drip.

**Video 1:** LAO caudal view, baseline angiogram of the left coronary arteries showing a tubular 90% stenosis in the proximal LAD artery and unremarkable LCx artery; also at https://youtube.com/shorts/BQNZMUK7jZ4. LAO: left anterior oblique; LAD: left anterior descending; LCx: left circumflex.

**Video 2:** LAO cranial view, baseline angiogram of the left coronary arteries showing no significant coronary artery disease in the mid to distal segments of the LAD and LCx coronary arteries; also at https://youtube.com/shorts/Cdq5XpylD_Y. LAO: left anterior oblique; LAD: left anterior descending; LCx: left circumflex.

**Video 3:** LAO view, baseline angiogram of the right coronary artery with no significant coronary artery disease; also at https://youtu.be/kqfThoyTGuI. LAO: left anterior oblique.

**Video 4:** RAO caudal view showing diffuse stenosis in the proximal to mid segment of the previously unremarkable LCx artery with TIMI 2 flow and diffuse stenosis in the previously normal mid to distal LAD segments with TIMI 0 flow, consistent with vasospasm; also at https://youtube.com/shorts/Da8UVUjPBik. RAO: right anterior oblique; LCx: left circumflex; TIMI: thrombolysis in myocardial infarction; LAD: left anterior descending.

**Figure 3:**
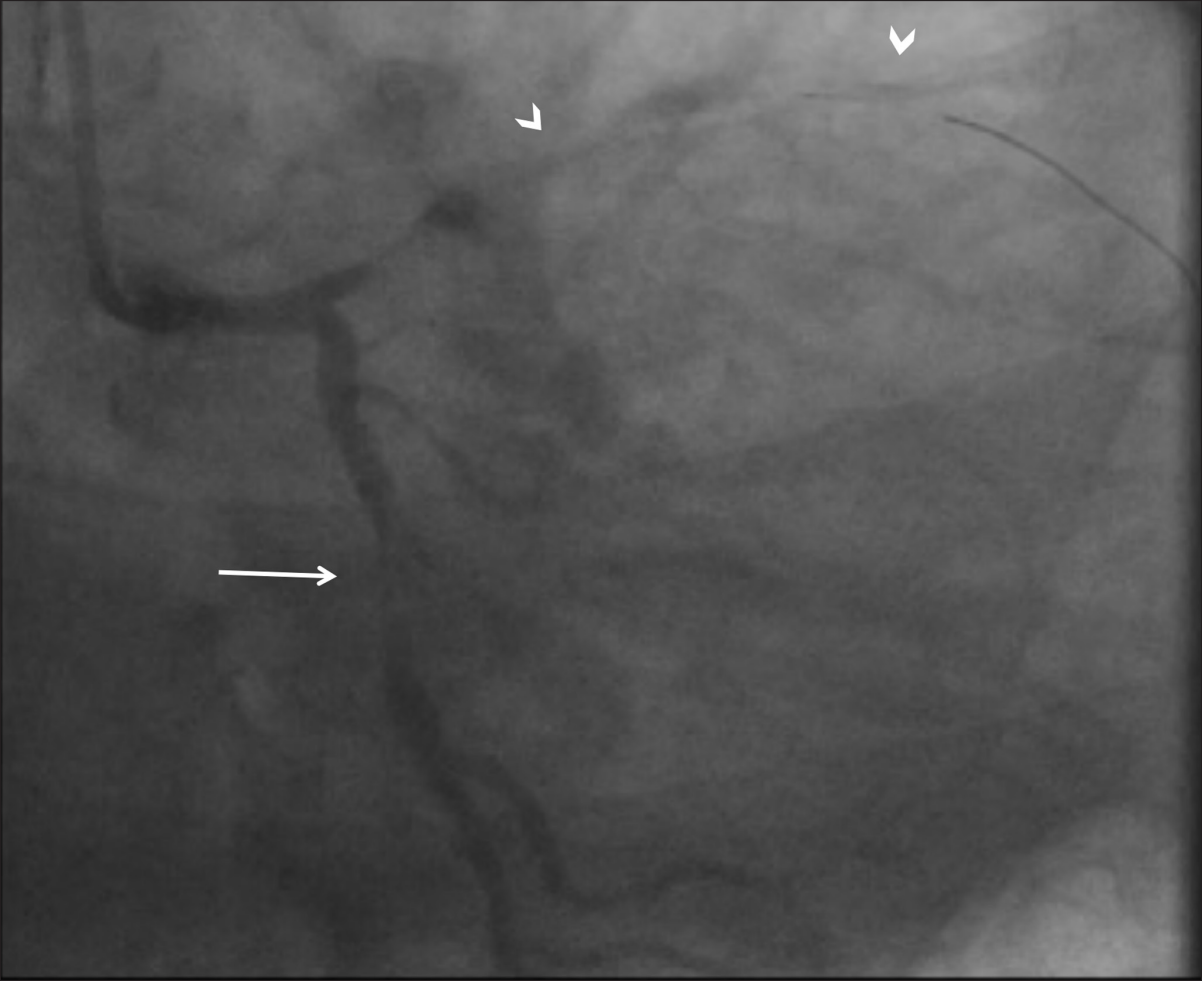
RAO caudal view showing diffuse stenoses in the LAD and LCx arteries with angiographically different segment involvement and severity. Compared to Figure 2, there is shorter segment involvement of the mid LCx artery (arrow) and more severe involvement of the mid to distal LAD artery (arrowheads). RAO: right anterior oblique; LAD: left anterior descending; LCx: left circumflex.

The initial impression was balloon-mediated dissection of the LAD artery with unclear etiology of LCx lesion. Subsequent angiograms reflected the transient yet recurrent nature of coronary lesions, which appeared to areas of vasospasm (Figure 3; Video 5). Immediate intervention of the proximal to mid LAD artery with two drug-eluting stents was performed to reestablish flow as quickly as possible.

**Video 5:** RAO caudal view showing diffuse stenoses in the LAD and LCx arteries with angiographically different segment involvement and severity. Compared to Figure 2 or Video 4, this shows a shorter segment involvement of the mid LCx artery and more severe involvement of the mid to distal LAD artery; also at https://youtube.com/shorts/80DlJSbQJB4. RAO: right anterior oblique; LAD: left anterior descending; LCx: left circumflex.

**Figure 4:**
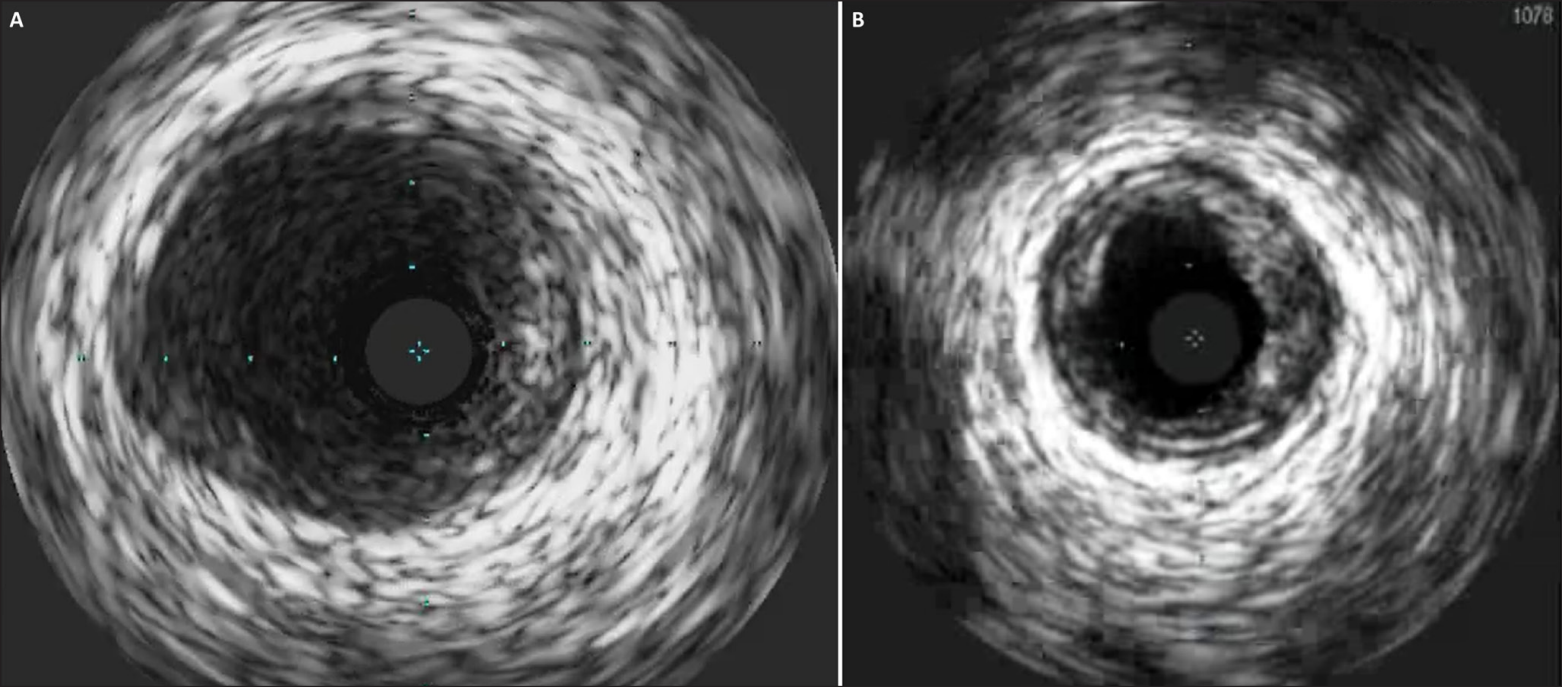
IVUS of the (**A**) left main coronary artery showing absence of dissection and (**B**) LCx artery showing absence of dissection with mild to moderate atherosclerotic disease. IVUS: intravascular ultrasound; LCx: left circumflex.

**Figure 5:**
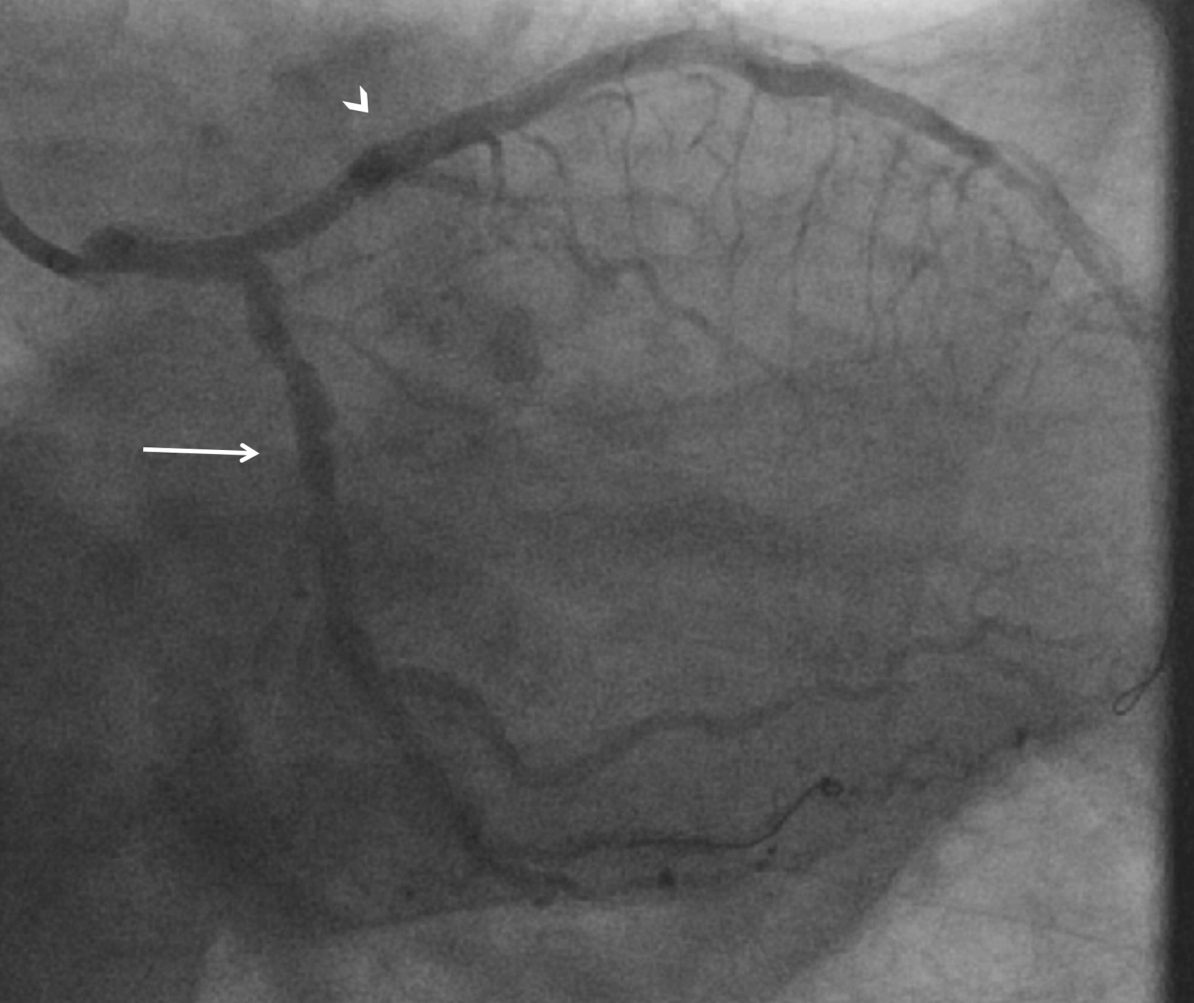
RAO caudal view showing resolution of LCx artery spasm (arrow) and presence of newly placed stents in the LAD artery (arrowhead) with TIMI 3 flow in both arteries. RAO: right anterior oblique; LCx: left circumflex; LAD: left anterior descending; TIMI: thrombolysis in myocardial infarction.

Considering the possibility of left main dissection with spiral extension into the LCx artery, we then performed intravascular ultrasound (IVUS) from the LCx and LAD arteries to the left main. While there was no evidence of left main or LCx artery dissection, IVUS did show areas of vasospasm in the LCx artery on repeat imaging and mild to moderate calcific atherosclerotic disease (Figure 4). When preparing for insertion of a temporary mechanical circulatory support device (Impella), we noticed a diffuse erythematous macular rash on the patient’s trunk. Because there was evidence of diffuse coronary vasospasm and diffuse rash, the presentation appeared consistent with an anaphylactic reaction. Administration of intravenous (IV) steroids, diphenhydramine, and IV fluids rapidly improved hemodynamics and symptoms, with resolution of ST elevation. Despite being a standard treatment for anaphylaxis, epinephrine was not given for risk of worsening coronary vasospasm. Repeat angiography showed complete resolution of coronary vasospasm and TIMI 3 flow in the LAD and LCx arteries (Figure 5; Video 6).

**Video 6:** Final result. RAO caudal view showing resolution of LCx artery spasm and presence of newly placed stents in the LAD artery with TIMI 3 flow in both arteries; also at https://youtube.com/shorts/BKSP7L-wXG0. RAO: right anterior oblique; LCx: left circumflex; LAD: left anterior descending; TIMI: thrombolysis in myocardial infarction.

## Discussion

Our patient presented with coronary vasospasm and shock in the setting of anaphylaxis due to contrast dye exposure during PCI. Acute coronary syndromes (ACS) due to coronary vasospasm and/or thrombosis occurring with allergic or anaphylactic reactions was initially described as a clinical entity in 1991 and has since been regarded as KS.^1^ There are three types of KS, all defined by allergic stimulation of proinflammatory (primarily mast-cell–mediated) and platelet activation pathways, which leads to local and systemic release of vasoactive and prothrombotic mediators and the consequent effects of their interrelationships. The three KS types include (type 1) coronary vasospasm in the setting of normal or near-normal coronary arteries in patients without predisposing factors for coronary artery disease reflecting endothelial dysfunction or microvascular disease, (type 2) coronary vasospasm with underlying quiescent atherosclerosis with or without plaque erosion or rupture, and (type 3) coronary stent thrombosis or in-stent restenosis. Of these, type 1 seems to be the most common.^1,2,3,4^

KS appears to be uncommon, but the incidence may be underestimated due to under-recognition.^2,3^ The reported incidence among patients presenting with suspected ACS undergoing urgent coronary angiography was 0.002%,^3^ 1.1% to 3.4% among patients presenting with allergic reactions,^2,5,6^ and around 0.19% among all hospitalizations.^6^ Post-marketing surveillance data from the US Food and Drug Administration Adverse Event Reporting System (FAERS) from 2009 to September 2020 show 1,083 reports of KS (out of more than 8.6 million reported adverse events over a similar timeframe), with 41 associated deaths.^7^ KS appears to be more common among males and those with underlying cardiovascular comorbidities.^4,5,7^ Medications, particularly nonsteroidal anti-inflammatory drugs, are the most common trigger for KS.^4,7^ As in our patient, contrast media has been the implicated trigger in almost 6% of reported KS cases.^4^

The diagnosis of KS is established clinically in a patient presenting with coronary vasospasm manifesting as acute coronary syndrome associated with allergic or anaphylactic signs and symptoms. In addition to the local coronary vascular effect, cardiovascular collapse such as persistent hypotension and shock can be perpetuated by concurrent systemic inflammatory or vasodilatory anaphylactic response with or without stress cardiomyopathy.^3^ Imaging studies also have added to our understanding of the pathophysiology of KS. Goto et al. demonstrated the presence of severe myocardial ischemia on SPECT imaging in a patient without significant coronary stenosis on angiography, consistent with vasospastic type 1 KS.^8^ A prospective study using cardiac magnetic resonance showed subendocardial perfusion defects and edema corresponding to ischemia and acute myocardial injury without evidence of irreversible myocardial damage in all patients who presented with KS, highlighting the potentially reversible nature of myocardial injury.^6^

Treatment of KS primarily involves managing the allergic/anaphylactic reaction. However, certain pharmacologic agents that prove beneficial for anaphylaxis may be harmful for coronary disease, and vice versa. For example, use of epinephrine for anaphylaxis may exacerbate coronary vasospasm and myocardial ischemia. Vasodilators and bolus administration of antihistamines should be used with caution as these can induce or worsen hypotension. Moreover, the use of opioids can further stimulate allergic reactions, so alternative analgesic or anxiolytic agents should be considered. In patients with significant coronary atherosclerotic lesions or occlusion, as with type 2 or 3 KS, traditional management of ACS should be employed.^3^

## Conclusion

KS is an uncommon yet life-threatening condition characterized by coronary vasospasm or thrombosis due to hypersensitivity reaction. A high index of suspicion is key to early diagnosis and initiation of appropriate treatment. Importantly, KS due to contrast dye should be strongly suspected during coronary angiography in cases of unexplained persistent or recurrent coronary vasospasm.
